# Addition to “All-Glass 100 mm Diameter Visible Metalens for Imaging the Cosmos”

**DOI:** 10.1021/acsnano.4c06432

**Published:** 2024-06-11

**Authors:** Joon-Suh Park, Soon Wei Daniel Lim, Arman Amirzhan, Hyukmo Kang, Karlene Karrfalt, Daewook Kim, Joel Leger, Augustine Urbas, Marcus Ossiander, Zhaoyi Li, Federico Capasso

**Figure 1:**
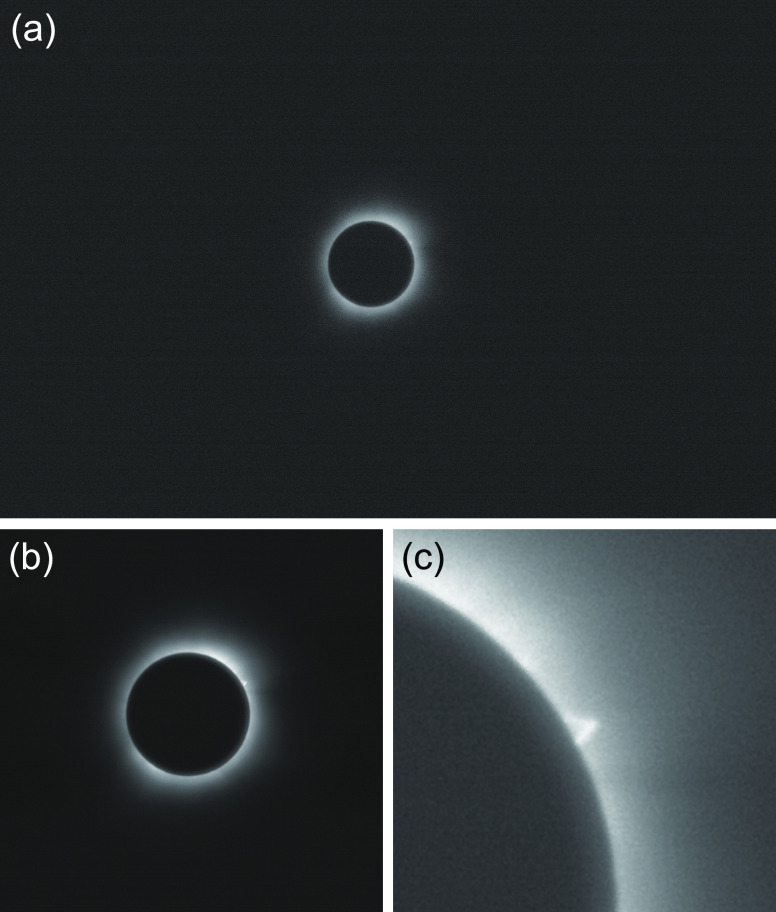
Raw and processed images of the 2024 total solar eclipse captured with the metalens. (a) A raw image of the solar eclipse at its totality that occurred on April 8th, 2024, taken with the meta-astrophotography apparatus. (b) Processed image of the solar eclipse after stacking five raw images of the eclipse using the SIRIL astronomical image processing tool and adjusting the contrast. The solar prominence is visible around the northeast edge of the Sun in the image. (c) Zoomed-in image of the solar prominence in (b). The full set of raw images and a processed image of the eclipse can be found at https://doi.org/10.6084/m9.figshare.25726533.

After our article was published in January 2024, a notable astronomical event occurred on April 8, 2024: a total solar eclipse traversed the North America region. One of the authors, Arman Amirzhan, captured the eclipse at its totality using the meta-astrophotography apparatus. In this Addition, we report the eclipse images obtained with the 100 mm diameter metalens. The meta-astrophotography apparatus was composed only of the 100 mm diameter metalens, a 1 nm bandwidth bandpass filter at λ = 632.8 nm (FL632.8-1, *Thorlabs*), and a cooled ZWO ASI183 mm-Pro CMOS sensor (monochrome, 13.2 mm × 8.8 mm sensor, 2.4 μm × 2.4 μm pixel pitch). The apparatus was mounted on a ZWO AM5 mount in altazimuth (Alt-AZ) configuration in solar tracking mode, without active guiding. Five raw images (Figure 1(a)) of the solar eclipse at totality were obtained with 10 ms exposure time per image in FITS format, with the CMOS sensor cooled to −21 °C, without pixel binning (Bin 1), and with zero gain. The imaging was performed near Irasburg, Vermont, USA at the following geographic coordinates: 44°49′50.5″N, 72°14′23.8″W. The collected images were stacked and processed using the SIRIL astronomical image processing tool (https://siril.org/), where each pixel value was averaged from 5 images to reduce the noise and improve the signal-to-noise ratio. The image contrast was adjusted using Adobe Photoshop (Figure 1(b)). A solar prominence,^2,3^ a bright plasma structure arching outward into the corona from the Sun’s photosphere, is visible at the northeast edge of the eclipse (Figure 1(c)). The unprocessed and processed eclipse images are available at https://doi.org/10.6084/m9.figshare.25726533.
